# Utilization of the iOS Shortcuts App to Generate a Surgical Logbook Tool: Feasibility Study

**DOI:** 10.2196/24644

**Published:** 2021-05-13

**Authors:** Daniel Thompson

**Affiliations:** 1 Department of Vascular Surgery St Vincent's Hospital Melbourne Fitzroy Australia

**Keywords:** app, audit, data collection, data, feasibility, medical education, mHealth, surgery, surgical audit, surgical education, utility

## Abstract

**Background:**

Surgical audit is an essential aspect of modern reflective surgical practice and is key to improving surgical outcomes. The surgical logbook is an important method of data collection for both personal and unit audits; however, current electronic data collection tools, especially mobile apps, lack the minimum recommended data fields.

**Objective:**

This feasibility study details the creation of a free, effective surgical logbook tool with the iOS Shortcuts app and investigates the time investment required to maintain a surgical logbook with this tool. In addition, we investigate the potential utility of the Shortcuts app in creating medical data collection tools.

**Methods:**

Using the iOS Shortcuts app, we created a shortcut “Operation Note,” which collects surgical logbook data by using the minimum and extended audit data sets recommended by the Royal Australasian College of Surgeons. We practically assessed the feasibility of the tool, assessing the time requirement for entry, accuracy, and completeness of the entered data.

**Results:**

The shortcut collected accurate and useful data for a surgical audit. Data entry took on average 65 seconds per case for the minimum data set, and 135 seconds per case for the extended data set, with a mean difference of 68 seconds (*P*<.001; 95% CI 61.6-77.7).

**Conclusions:**

This feasibility study demonstrates the utility of the iOS Shortcuts app in the creation of a surgical logbook and the time-consuming nature of data collection for surgical audit. Our iOS Operation Note shortcut is a free, rapid, and customizable alternative to currently available logbook apps and offers surgical trainees and consultants a method for recording surgical operations, complications, and demographic data.

## Introduction

Surgical audit is one of the mainstays of reflective practice and improving patient outcomes. Audit allows for the capture of operative results, operation numbers, billing information, and complications. In Australia, it is mandatory to maintain a logbook when undertaking surgical training with the Royal Australasian College of Surgeons (RACS); RACS provides a minimum and extended suggested data set for both trainee and consultant logbooks [1].

Owing to the increasing complexity of health care, electronic information systems are recommended to be utilized to capture clinical data. The use of hospital medical record systems relies heavily on accurate data being captured by staff and may not include the required fields or enough detail to facilitate successful audit of practice [1]. Hence, RACS recommends that surgeons maintain personal logbooks [1]. Many surgeons utilize technology, such as mobile phones, to collect these data; however, on recent review, the applications available in the Australian market fall short of both the suggested minimum and extended data set, highlighting the need for a better surgical logbook tool [2].

The most commonly reported barrier to successful logbook-keeping is the time required for data entry and maintenance [3]. A key difficulty is navigating the balance between increased data capture for more complete and useable information and the extended amount of time required to enter such data. Data overload is “a danger to a successful audit” and this balance must be carefully considered when designing an audit tool [1]. The RACS minimum data set consists of 12 data points, and the extended data set contains 24 data points [1].

In Australia, the primary surgical audit tool for trainees and consultants is the Morbidity Audit and Logbook Tool (MALT) and is mandatory for surgical trainees in certain colleges [4]. MALT contains both the minimum and extended data set and is a useful tool in audit and reflective practice. However, there are some barriers to mobile access to MALT, including a multistep login process, reliance on an internet connection, and the absence of a mobile app, which may limit its use in prospective data capture [2].

It is unclear what proportion of surgeons prospectively maintain a logbook versus those who carry out a retrospective review prior to a regular audit. Prospective record-keeping has the added advantage of being more accurate and resolves issues regarding the evaluation of operative volumes due to computerized or paper-based medical record systems, which may be missed if coded incorrectly, or in procedures with multiple surgeons or specialties [5]. Mobile apps offer an accessible, rapid, and easy-to-use method of prospective record keeping.

Electronic data capture tools have been widely adopted by researchers in medicine and provide an effective way of prospectively collecting data for storage and tabulation [6]. More than 100 different data capture tools are currently available, ranging from those specifically designed for capturing clinical data, such as REDCap, to informal survey software, such as SurveyMonkey and Google Forms [7]. Formal data collection tools such as REDCap and Open Data Kit are usually compliant with established national data security regulations; however, they often have a requirement for some knowledge of computer programming, with an associated learning curve [8]. Nonclinical tools such as SurveyMonkey and Google Forms require an active internet connection and lack end-to-end encryption [8]. Despite these concerns, many institutions have utilized simple data capture tools owing to the simplicity of their implementation and their cost-effectiveness [9].

While RACS provides a guideline regarding the minimum and extended criteria required for a logbook, certain surgeons and specialties may benefit from customization, allowing for further categorization of operative data for personal or research purposes and more efficient data entry [3].

The iOS Shortcuts app (hereinafter referred to as “Shortcuts”) was released in 2019 and allows users to create custom macros allowing for data entry, manipulation, and storage on any iOS-compatible devices [10]. Rather than computer programming, Shortcuts provides the user with a list of tasks to select, which are then customized and transformed to a workflow to be activated by the user. The software utilizes end-to-end encryption when transmitting data and is stored on an encrypted and secure iCloud server.

There is currently no literature describing the potential use of Shortcuts in the collection of clinical data. This feasibility study highlights the utility of the application and demonstrates its functionality in the creation of a surgical logbook tool.

## Methods

A custom shortcut was designed using the freely available iOS Shortcuts app on an iPad Pro (11-inch) device running on iOS 13.7.

A Numbers (Apple, Cupertino) Spreadsheet document was created in a suitable folder of the iPad storage with the address “/Shortcuts/Logbook/Logbook.numbers” (Multimedia Appendix 1). A sheet and table-titled logbook were created (Multimedia Appendix 1, Sheet 1). An additional file and table were created for the extended data sheet (Multimedia Appendix 2). These files have been provided as a Microsoft Excel spreadsheet to facilitate review from both Windows and Apple devices; however, the shortcut tool requires the Apple Numbers file format to operate successfully.

**Figure 1:**
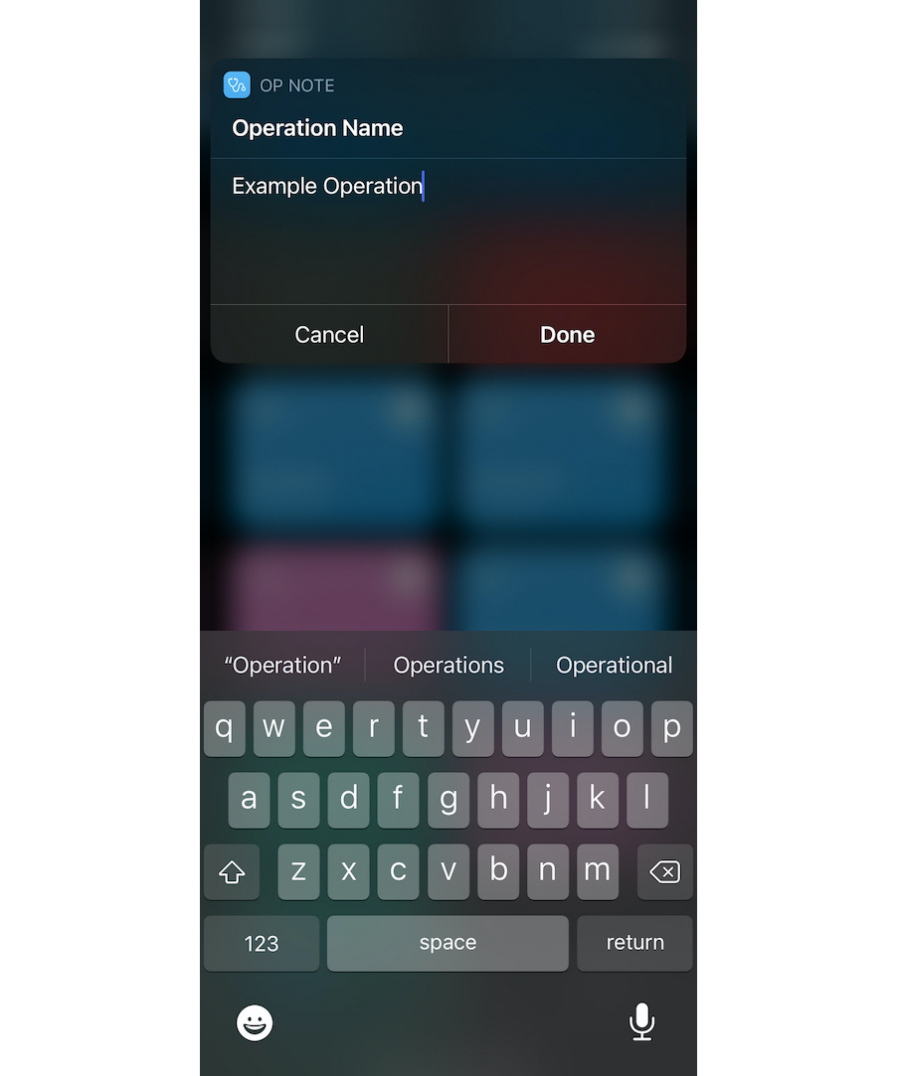
Example of a shortcut in operation with a user prompt to enter the operation name.

**Figure 2:**
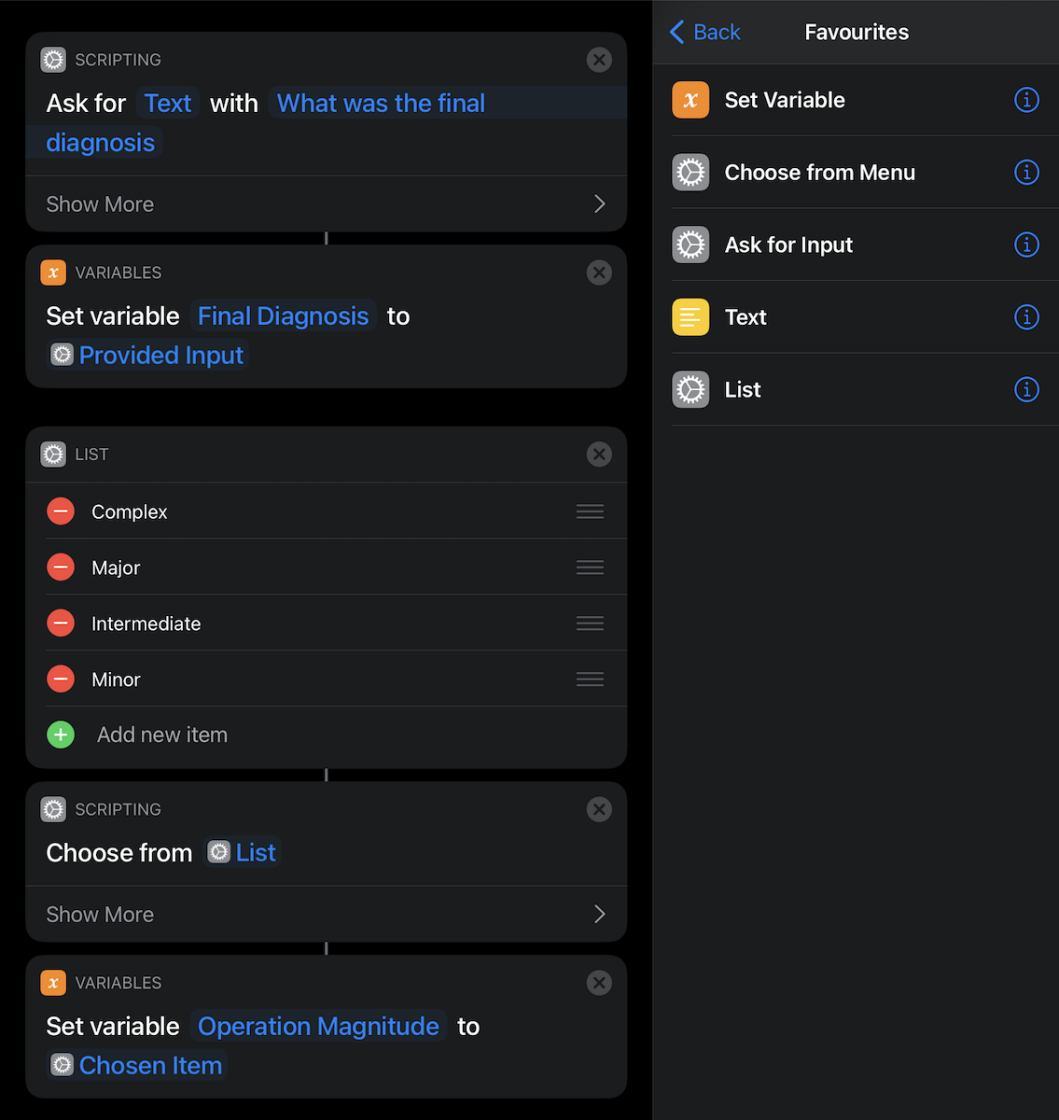
Workflow, free text, and "Choose from list" data entry shortcuts in the iOS Shortcuts app.

**Figure 3:**
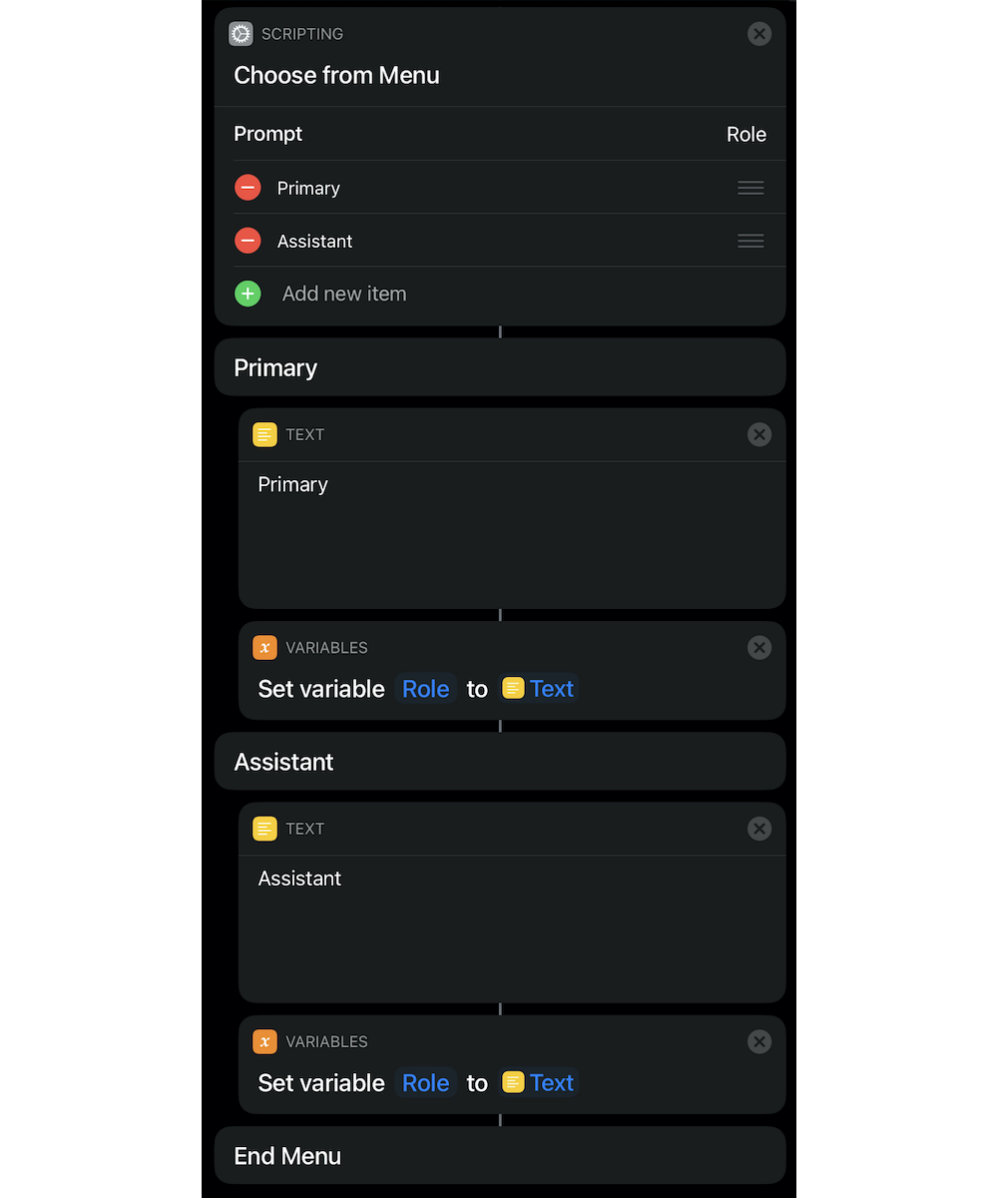
Workflow and "Choose from menu" data entry shortcut in the iOS Shortcuts app.

Using Shortcuts, we generated a workflow macro for the logbook. The workflow consists of multiple data entry blocks, consisting of a prompt for the user with a question, followed by the appearance of a text box accepting the user-entered data, and finally storage as a variable (Figure 1). There are three ways this system is used: a free text entry (Figure 2) and 2 menu choice operations; that is, list or menu (Figures 2 and 3). An agile software development approach was utilized with iterative design, development, testing, and refinement [11].

In total, 2 shortcuts were designed with 2 separate receiving spreadsheets, first with the minimum RACS recommended data set, both of which are freely available through the iCloud service [12,13].

For the purpose of evaluation, a timer was started at the initiation of the shortcut and stopped upon data storage, in order to determine the time taken to use this tool. This timer was excluded for the provided example shortcuts and spreadsheets.

Upon conclusion of the shortcut, the Numbers spreadsheet was opened, and the listed variables were stored as a new row with variables entered into each subsequent column.

A second spreadsheet was created to demonstrate the analytical capability of the logbook tool for audit purposes. This example data analysis tool is provided in Multimedia Appendix 1 (Sheet 2). Prefilled formulas were created for case numbers, operation categories, patient gender, complications and grades, and length of stay, and automatically updated as new cases were entered.

The tool was practically assessed for feasibility, with a surgical registrar entering 20 mock operations into the minimum and extended data field shortcuts. The operation notes were created randomly by computer software with a selection of common operations, indications, and demographic data. Feasibility was assessed by assessing the time required for data entry, the completeness of the entered data, and the accuracy of the filled spreadsheet with the required clinical data.

## Results

Shortcuts performed well during testing and has been shown to collect both complete and useful data. The average data entry time for an operation across both minimum and extended data sets was 100 seconds.

In total, 20 cases were recorded with the minimum and extended data sets. The average time for completion of the minimum data set was 65 seconds, with a total time of 21 minutes 43 seconds for 20 cases. The average time for completion of the extended data set was 135 seconds, with a total time of 46 minutes 58 seconds for 20 cases. There was a mean difference of 68 seconds per case between the minimum and extended data sets compared to the minimum data set (*P*<.001; 2-tailed *t_19_*=17.52; 95% CI 61.6-77.7). Table 1 summarizes the time requirements for data entry with the Shortcut.

**Table 1:** Time requirement for data entry in the minimum and extended data sets.

Week	Cases (n)	Average time to complete data entry (seconds)	Total time spent entering cases, minutes (seconds)
1 (minimum data set)	20	65	21 (43)
2 (extended data set)	20	135	46 (58)

The built-in example data analysis table functioned well, having captured the entered data and presented basic statistical analysis in real time. Multimedia Appendix 1 (Sheet 2) summarizes the data fields used. On analyzing the spreadsheet, 100% of the required fields were filled successfully, since the shortcut design does not allow incomplete data to be entered.

## Discussion

### Principal Findings

Surgical audit is key for modern successful surgical practice, allowing for reflection on case numbers, outcomes, complications, and deaths. A surgical logbook is a key tool for collecting these data and can be utilized in both personal and unit audits. Surgeons are increasingly turning to technology to complete tasks more efficiently and accurately; however, mobile logbook offerings in the Australian setting still do not meet the recommended minimum requirements [2]. The main barrier for the completion of a prospective surgical logbook is the time required for completion, and mobile apps hold the potential for a tool that is portable, accurate, and time saving.

The most commonly reported barrier to the maintenance of a surgical logbook is time [1]. Collection of such a wide range of data points, as exemplified by the extended data set, requires an easy-to-use, rapid, and efficient tool to accurately record data in a timely manner. Retrospective data collection may be less accurate owing to time elapsed since the operation date, and automatic audit from hospital-recorded data may not include all required fields. Our tool provides an accurate, customizable system for collecting audit data.

MALT is a surgical auditing tool available to trainees and consultants as part of their college membership and is also available to resident medical officers as part of the JDocs Framework of the RACS (costing AU $345=US $268.14 annually) [14]. This cost may be a barrier to its use. This is especially relevant as previous studies have demonstrated that residents who complete a logbook are more likely to complete surgical procedures; as such, an accessible and free logbook app (eg, Shortcuts developed herein) may be of use in junior physicians’ reflective practice and potentially increase their motivation to be involved in the operating theater [5].

Ahmadi et al [2] explored a number of available logbook tools in their recent review and reported that none of them collected sufficient data to meet the minimum and extended data sets recommended by the RACS. Shortcuts collects all required fields for either the minimum or extended data sets. While custom data collection tools such as REDCap and Open Data Kit could be utilized to create a surgical logbook tool, they would require extensive coding and app development skills to create a mobile interface [15].

Our app was created without the use of code, utilizing Shortcut’s “drag and drop” interface, which allowed for accessible and easy creation of a data collection tool on a mobile device. This software is well suited for an app such as the surgical logbook, and the provided example files can be utilized as a framework to create a specialty-specific logbook with user customization. Our shortcut program is freely available on the internet [12,13] and can be used quickly after installation (Multimedia Appendices 1 and 2) in a Numbers spreadsheet file format.

Shortcuts has been designed to allow for easy end user modification, and it provides a framework from which surgeons, trainees, and residents can create a surgical logbook that suits their needs. For example, in vascular surgery, a categorical field may be added for arterial, venous, or renal access fields, as found in the Australasian Vascular Audit [16]. The tool allows for audit data to be generated from a spreadsheet, like with many other data collection tools, and users can design their own analysis spreadsheet to provide relevant summary statistics and graphs. An example of basic statistical analysis is provided in Multimedia Appendix 1 (Sheet 2), which allows for rapid review of a number of summary statistics.

Further improvements to our app could include integration with a web-based system such as MALT, importing and saving operation notes for future reference, and barcode scanning for universal record number entries in hospitals that use compatible barcode labels. At present, no logbook apps that allow integration with MALT are available [2].

### Limitations

A primary limitation of this tool in some jurisdictions is the use of iCloud storage. This is necessitated by the design limits of Shortcuts, which, at present, prevents local iOS storage. This limitation is shared among many currently available apps and is a challenge for mobile data collection tools in general; these tools often have advanced security features but lack official data regulation accreditation [17-19]. The iCloud service is highly secure, requiring 2-factor authentication and utilizing end-to-end encryption; these features are not shared by more informal survey tools such as SurveyMonkey. iCloud’s security potentially meets the regulatory specifications for protocols such as the Health Insurance Portability and Accountability Act or General Data Protection Regulation; however, the lack of signed industrial agreements has limited formal accreditation [20].

Australia does not have a formal health information act such as Health Insurance Portability and Accountability Act or General Data Protection Regulation; rather, organizations and clinicians are required to take “reasonable steps” to protect the privacy of patient data [19]. Accordingly, the RACS MALT service utilizes 2048-bit key encryption to secure connections but has no formal data protection regulatory agreements in place [21]. Shortcuts potentially meets the minimum standards for data protection; however, it is the responsibility of users to ensure that data are collected in line with the data protection regulations within their jurisdictions.

Another limitation of both this app and other audit tools, including MALT, is that prospectively entering operation details at the time of the operation may result in nonrecording of delayed complications [1]. Shortcuts allows for immediate postoperative complications to be entered, such as hemorrhage or death; however, complications that occur in the days and weeks following an operation need to be entered manually and retrospectively upon their occurrence, in the data spreadsheet. Further development of this app and the inclusion of an additional complications shortcut may allow for automation of data entry related to complications.

### Conclusions

This study shows the feasibility of utilizing the iOS Shortcuts app as a data collection tool, as revealed through the creation of a surgical logbook. Shortcuts is highly customizable and has a wide range of potential applications including data collection; moreover, it can be used as an interactive medical algorithm tool that allows for the creation of clinical interaction guidelines based on user input in the future.

## Appendix

Multimedia Appendix 1 Minimum data set spreadsheet.

Multimedia Appendix 2 Extended data set spreadsheet.

## Abbreviations

MALT: Morbidity Audit and Logbook Tool
RACS: Royal Australasian College of Surgeons
